# Role of Artificial Intelligence Applications in Real-Life Clinical Practice: Systematic Review

**DOI:** 10.2196/25759

**Published:** 2021-04-22

**Authors:** Jiamin Yin, Kee Yuan Ngiam, Hock Hai Teo

**Affiliations:** 1 Department of Information Systems and Analytics School of Computing National University of Singapore Singapore Singapore; 2 Department of Surgery National University Hospital Singapore Singapore

**Keywords:** artificial intelligence, machine learning, deep learning, system implementation, clinical practice, review

## Abstract

**Background:**

Artificial intelligence (AI) applications are growing at an unprecedented pace in health care, including disease diagnosis, triage or screening, risk analysis, surgical operations, and so forth. Despite a great deal of research in the development and validation of health care AI, only few applications have been actually implemented at the frontlines of clinical practice.

**Objective:**

The objective of this study was to systematically review AI applications that have been implemented in real-life clinical practice.

**Methods:**

We conducted a literature search in PubMed, Embase, Cochrane Central, and CINAHL to identify relevant articles published between January 2010 and May 2020. We also hand searched premier computer science journals and conferences as well as registered clinical trials. Studies were included if they reported AI applications that had been implemented in real-world clinical settings.

**Results:**

We identified 51 relevant studies that reported the implementation and evaluation of AI applications in clinical practice, of which 13 adopted a randomized controlled trial design and eight adopted an experimental design. The AI applications targeted various clinical tasks, such as screening or triage (n=16), disease diagnosis (n=16), risk analysis (n=14), and treatment (n=7). The most commonly addressed diseases and conditions were sepsis (n=6), breast cancer (n=5), diabetic retinopathy (n=4), and polyp and adenoma (n=4). Regarding the evaluation outcomes, we found that 26 studies examined the performance of AI applications in clinical settings, 33 studies examined the effect of AI applications on clinician outcomes, 14 studies examined the effect on patient outcomes, and one study examined the economic impact associated with AI implementation.

**Conclusions:**

This review indicates that research on the clinical implementation of AI applications is still at an early stage despite the great potential. More research needs to assess the benefits and challenges associated with clinical AI applications through a more rigorous methodology.

## Introduction

### Background

Artificial intelligence (AI) has greatly expanded in health care in the past decade. In particular, AI applications have been applied to uncover information from clinical data and assist health care providers in a wide range of clinical tasks, such as disease diagnosis, triage or screening, risk analysis, and surgical operations [1-4]. According to Accenture analysis, the global health AI market is expected to reach US $6.6 billion by 2021 and has the potential to grow more than 10 times in the next 5 years [5].

The term “AI” was coined by McCarthy in the 1950s and refers to a branch of computer science wherein algorithms are developed to emulate human cognitive functions, such as learning, reasoning, and problem solving [6]. It is a broadly encompassing term that includes, but is not limited to, machine learning (ML), deep learning (DL), natural language processing (NLP), and computer vision (CV).

Researchers have devoted a great deal of effort to the development of health care AI applications. The number of related articles in the Google Scholar database has grown exponentially since 2000. However, their implementation in real-life clinical practice is not widespread [1,7]. Several reasons may account for this research-practice gap. Specifically, AI algorithms may be subject to technical issues, such as data set shift, overfitting, bias, and lack of generalizability [8], limiting the safe translation of AI research into clinical practice. Further, practical implementation of AI applications can be incredibly challenging. Examples of key challenges that need to be addressed include data sharing and privacy issues, lack of algorithm transparency, the changing nature of health care work, financial concerns, and the demanding regulatory environment [1,3,9-13]. However, the huge potential of health care AI applications can only be realized when they have been integrated into clinical routine workflows.

### Research Gap

To the best of our knowledge, this review is the first to systematically examine the role of AI applications in real-life clinical environments. We note that many reviews have been carried out in the area of health care AI. One stream of reviews provided an overview of the current status of AI technology in specific clinical domains, such as breast cancer diagnosis [14], melanoma diagnosis [15], pulmonary tuberculosis diagnosis [16], stroke diagnosis and prediction [17], and diabetes management [18]. Another stream of reviews focused on comparing clinician performance and AI performance to provide the evidence base needed for AI implementation [19-21]. In contrast, our work differs from previous reviews in at least three aspects. First, we review clinical AI applications that provide decision support more broadly and hence do not restrict our scope to a specific clinical domain. Second, we focus on studies that reported the evaluation of clinical AI applications in the real world. We hence exclude studies that discussed the development and validation of clinical AI algorithms without actual implementation. Finally, we report a wide range of evaluation outcomes associated with AI implementation, such as performance comparison, clinician and patient outcomes, and economic impact.

On the other hand, we note that several viewpoint articles have provided a general outlook of health care AI [1-3,7,9,22]. These articles mainly provided insights into the current status of health care AI and selected a few clinical AI applications as illustrative examples. They might have also discussed the challenges associated with the practical implementation of AI. However, these articles did not discuss the progress of AI implementation that had been made in detail. In contrast, our work aims to provide a comprehensive map of the literature on the evaluation of AI applications in real-life clinical settings. By doing so, we summarize empirical evidence of the benefits and challenges associated with AI implementation and provide suggestions for future research in this important and promising area.

### Objective

The objective of this systematic review was to identify and summarize the existing research on AI applications that have been implemented in real-life clinical practice. This helps us better understand the benefits and challenges associated with AI implementation in routine care settings, such as augmenting clinical decision-making capacity, improving care processes and patient outcomes, and reducing health care costs. Specifically, we synthesize relevant studies based on (1) study characteristics, (2) AI application characteristics, and (3) evaluation outcomes and key findings. Considering the research-practice gap, we also provide suggestions for future research that examines and assesses the implementation of AI in clinical practice.

## Methods

### Search Strategies

The systematic review was conducted following the Preferred Reporting Items for Systematic Reviews and Meta-Analyses (PRISMA) guidelines [23]. We searched PubMed, Embase, Cochrane Central, and CINAHL in June 2020 to identify relevant articles on AI applications that had been implemented in clinical practice. We limited our search to English-written peer-reviewed journal articles published between January 2010 and May 2020. We chose 2010 as the start period because health care AI research has since taken off.

We used two groups of keywords to identify terms in the titles, abstracts, and keywords of the publications. The first group of keywords had AI-related terms, including “artificial intelligence,” “machine learning,” and “deep learning.” It is worth noting here that AI is a broadly encompassing term and also includes specific AI techniques, such as neural networks, support vector machines, decision trees, and NLP. However, studies using these techniques are highly likely to use “artificial intelligence” or “machine learning” in abstracts or keywords [24]. The second group of keywords had terms related to clinical implementation, including “clinical,” “health,” “healthcare,” “medical,” “implement,” “implementation,” “deploy,” “deployment,” and “adoption.” Details of the search strategy can be found in Multimedia Appendix 1.

### Eligibility Criteria

We downloaded and imported all of the identified articles using EndNote X9 (Thomson Reuters) for citation management. After removing duplicates, two researchers (JY and KYN) independently screened the titles and abstracts of the identified articles to determine their eligibility. Disagreements were resolved by discussion between the authors until consensus was reached. The inclusion criteria were as follows: (1) the study implemented an AI application with patients or health care providers in a real-life clinical setting and (2) the AI application provided decision support by emulating clinical decision-making processes of health care providers (eg, medical image interpretation and clinical risk assessment). Medical hardware devices, such as X-ray machines, ultrasound machines, surgery robots, and rehabilitation robots, were outside our scope.

The exclusion criteria were as follows: (1) the study discussed the development and validation of clinical AI algorithms without actual implementation; (2) the AI application provided automation (eg, automated insulin delivery and monitoring) rather than decision support; and (3) the AI application targeted nonclinical tasks, such as biomedical research, operational tasks, and epidemiological tasks. We also excluded conference abstracts, reviews, commentaries, simulation papers, and ongoing studies.

### Data Extraction and Charting

Following article selection, we created a data-charting form to extract information from the included articles in the following aspects: (1) study characteristics, (2) AI application characteristics, and (3) evaluation outcomes and key findings (Textbox 1).

**Components of the data-charting form.**

**Study characteristics**

- Author, year
- Study design
- Involved patient(s) and health care provider(s)
- Involved hospital(s) and country of the study

**Artificial intelligence (AI) application characteristics**

- Application description
- AI techniques used (eg, neural networks, random forests, and natural language processing)
- Targeted clinical tasks
- Targeted disease domains and conditions

**Evaluation outcomes and key findings**

- Performance of AI applications
- Clinician outcomes
- Patient outcomes
- Cost-effectiveness

## Results

### Overview

**Figure 1:**
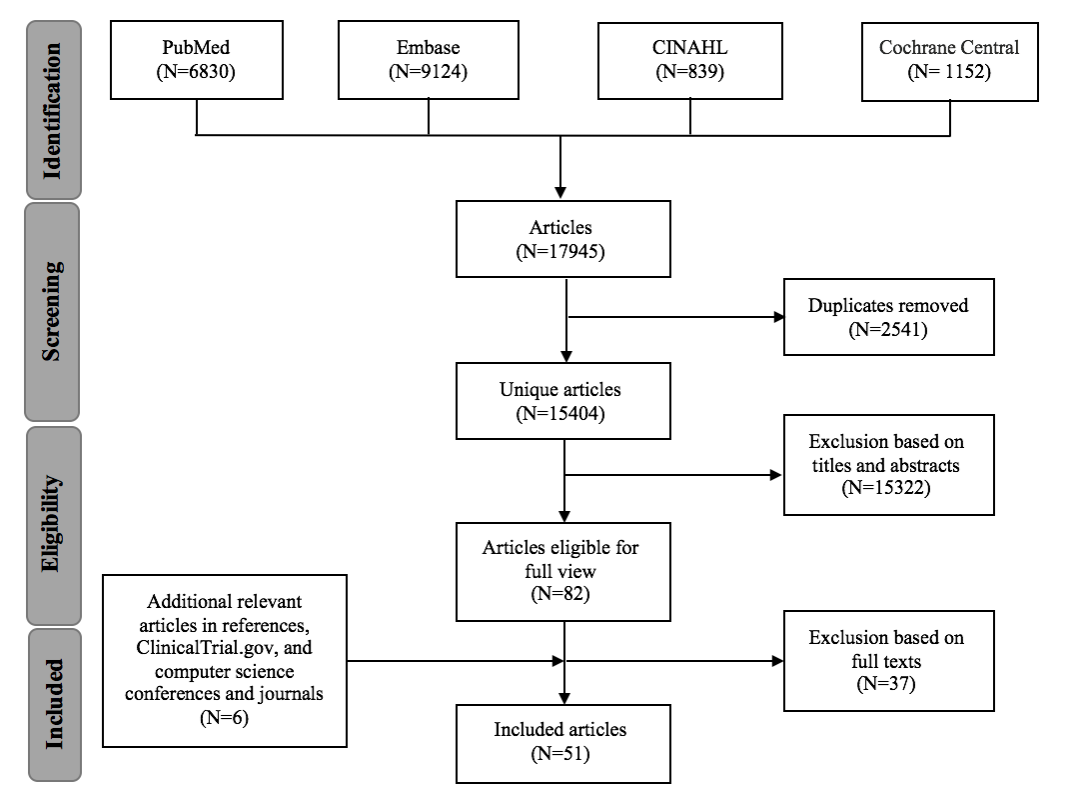
Flow diagram of the literature search based on the Preferred Reporting Items for Systematic Reviews and Meta-Analyses (PRISMA) statement.

Our initial search in June 2020 returned a total of 17,945 journal articles (6830 from PubMed, 9124 from Embase, 839 from CINAHL, and 1152 from Cochrane Central) (Figure 1). We first identified and excluded 2541 duplicates. After that, we excluded 15,322 articles after screening the titles and abstracts. Thus, 82 articles remained for full-text review, of which 45 were included in this review. Additionally, we identified six relevant articles by examining the references of the included articles, browsing through ClinicalTrial.gov using AI-related keywords, and hand searching premier computer science journals and conferences in AI (Multimedia Appendix 1). Finally, a total of 51 articles met our inclusion criteria.

### Study Characteristics

**Figure 2:**
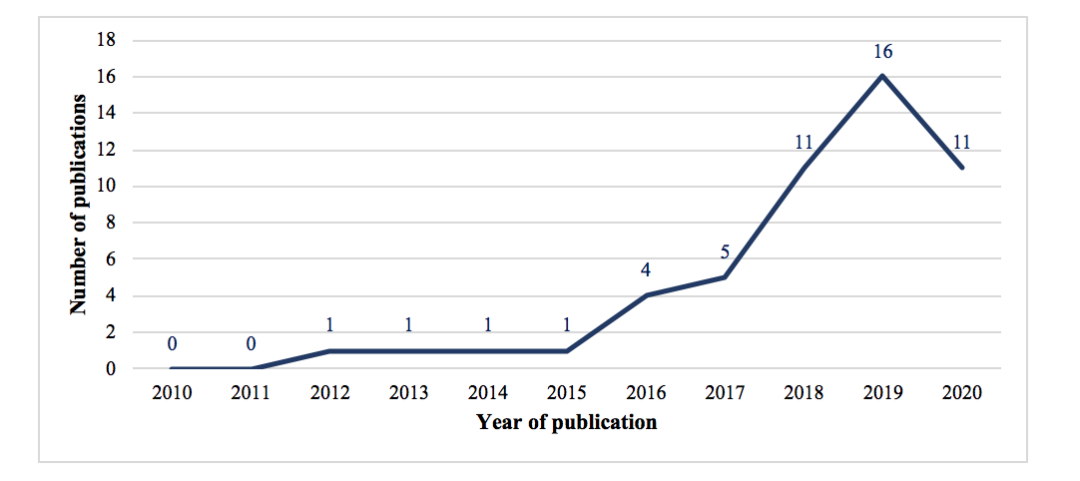
Distribution of the included articles from 2010 to 2020.

Table 1 summarizes the authors, year of publication, study design, involved patients and health care providers, and involved hospitals [25-75]. As shown in Figure 2, there was a rising trend in the number of included studies in the last decade, with a recent peak in 2019, suggesting accelerated research activity in this area.

Regarding study design, the 51 studies included 20 observational studies (17 prospective studies and three retrospective studies), 13 randomized controlled trials (RCTs), eight experimental studies, four before-and-after studies, three surveys, one randomized crossover trial, one nonrandomized trial, and one structured interview. It is important to note that observational studies can be categorized into prospective and retrospective studies based on the timing of data collection. In prospective studies, researchers design the research and plan the data collection procedures before any of the subjects have the disease or develop other outcomes of interest. In retrospective studies, researchers collect existing data on current and past subjects, that is, subjects may have the disease or develop other outcomes of interest before researchers initiate research design and data collection.

Of the 51 studies, 29 (57%) explicitly mentioned the involved patients, two of which had a sample size smaller than 30. On the other hand, 28 (55%) studies provided information about the involved health care providers, of which 17 studies had 10 or fewer providers.

**Figure 3:**
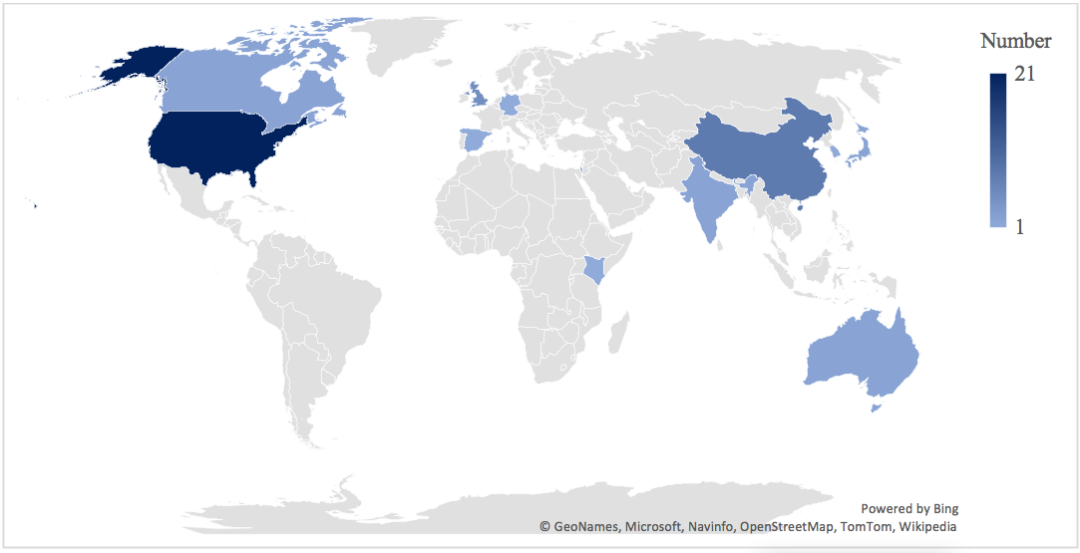
Country distribution of the involved hospitals.

Additionally, 46 (90%) studies mentioned the involved hospitals or clinics (Figure 3). Of these, 36 studies were conducted in developed countries, with 20 conducted in the United States, five in the United Kingdom, two each in Australia, Canada, and Japan, one each in Germany, Israel, Spain, and the Netherlands, and one in the United States and South Korea. On the contrary, 10 studies were conducted in developing countries, with eight conducted in China, one in India, and one in India and Kenya.

**Table 1:** Characteristics of the included studies.

Author, year	Study design	Sample characteristics	Hospital (country)	Evaluation outcomes
Abràmoff et al, 2018 [25]	Observational study (prospective)	819 patients	10 primary care clinics (United States)	AP^a^ (sensitivity, specificity, imageability rate)
Aoki et al, 2020 [26]	Experimental study (cross-over design)	6 physicians	The University of Tokyo Hospital (Japan)	CO^b^ (reading time, mucosal break detection rate)
Arbabshirani et al, 2018 [27]	Observational study (prospective)	347 routine head CT^c^ scans of patients	Geisinger Health System (United States)	AP (AUC^d^, accuracy, sensitivity, specificity) CO (time to diagnosis)
Bailey et al, 2013 [28]	Crossover RCT^e^	20,031 patients	Barnes-Jewish Hospital (United States)	PO^f^ (ICU^g^ transfer, hospital mortality, hospital LOS^h^)
Barinov et al, 2019 [29]	Experiment (within subjects)	3 radiologists	NR^i^	AP (AUC) CO (diagnostic accuracy)
Beaudoin et al, 2016 [30]	Observational study (prospective)	350 patients (515 prescriptions)	Centre hospitalier universitaire de Sherbrooke (Canada)	AP (number of triggered recommendations, precision, recall, accuracy)
Bien et al, 2018 [31]	Experimental study (within subjects)	9 clinical experts	Stanford University Medical Center (United States)	AP (AUC) CO (specificity, sensitivity, accuracy)
Brennan et al, 2019 [32]	Nonrandomized trial	20 physicians	An academic quaternary care institution (United States)	AP (AUC) CO (risk assessment changes, AUC, usability)
Chen et al, 2020 [33]	RCT	437 patients	Renmin Hospital, Wuhan University (China)	CO (blind spot rate)
Connell et al, 2019 [34]	Before-after study	2642 patients	Royal Free Hospital, Barnet General Hospital (United Kingdom)	PO (renal recovery rate, other clinical outcomes, care process)
Eshel et al, 2017 [35]	Observational study (prospective)	6 expert microscopists	Apollo Hospital, Chennai (India); Aga Khan University Hospital (Kenya)	AP (sensitivity, specificity, species identification accuracy, device parasite count)
Giannini et al, 2019 [36]	Before-after study	22,280 patients in the silent period, 32,184 patients in the alert period	3 urban acute hospitals under University of Pennsylvania Health System (United States)	AP (sensitivity, specificity) PO (mortality, discharge disposition, ICU transfer, time to ICU transfer, clinical process measures)
Ginestra et al, 2019 [37]	Survey	43 nurses and 44 health care providers	A tertiary teaching hospital in Philadelphia (United States)	CO (nurse and provider perceptions)
Gómez-Vallejo et al, 2016 [38]	Observational study (retrospective)	1800 patients (2569 samples)	A Spanish National Health System hospital (Spain)	AP (accuracy) CO (system perceptions)
Grunwald et al, 2016 [39]	Observational study (retrospective)	15 patients, 3 neuroradiologists	A comprehensive stroke center (Germany)	AP (e-ASPECTS performance)
Kanagasingam et al, 2018 [40]	Observational study (prospective)	193 patients, 4 physicians	A primary care practice in Midland (Australia)	AP (sensitivity, specificity, PPV^j^, NPV^k^)
Keel et al, 2018 [41]	Survey	96 patients	St Vincent’s Hospital, University Hospital Geelong (Australia)	AP (sensitivity and specificity, assessment time) PO (patient acceptability)
Kiani et al, 2020 [42]	Experimental study (within subjects)	11 pathologists	Stanford University Medical Center (United Kingdom)	AP (accuracy) CO (accuracy)
Lagani et al, 2015 [43]	Observational study (prospective)	2 health care providers	Chorleywood Health Centre (United Kingdom)	AP (system performance) CO (usability)
Lin et al, 2019 [44]	RCT	350 patients	5 ophthalmic clinics (China)	AP (accuracy, PPV, NPV) CO (time to diagnosis) PO (patient satisfaction)
Lindsey et al, 2018 [45]	Experimental study (within subjects)	40 practicing emergency clinicians	Hospital for Special Surgery (United States)	AP (AUC) CO (sensitivity, specificity, misinterpretation rate)
Liu et al, 2020 [46]	RCT	1026 patients	No. 988 Hospital of Joint Logistic Support Force of PLA (China)	CO (ADR^l^, PDR^m^, number of detected adenomas and polyps)
Mango et al, 2020 [47]	Experimental study (within subjects)	15 physicians	13 different medical centers (United States)	AP (AUC, sensitivity, specificity) CO (AUC, interreliability, intrareliability)
Martin et al, 2012 [48]	Observational study (prospective)	214 patients	13 different medical centers (United States)	AP (sensitivity, PPV) PO (ACSC^n^, care-supported activities)
McCoy and Das, 2017 [49]	Before-after study	1328 patients	Cape Regional Medical Center (United States)	PO (hospital mortality, hospital LOS, readmission rate)
McNamara et al, 2019 [50]	Observational study (prospective)	3 breast cancer experts	John Theurer Cancer Center (United States)	CO (decision making)
Mori et al, 2018 [51]	Observational study (prospective)	791 patients, 23 endoscopists	Showa University Northern Yokohama Hospital (Japan)	AP (NPV) CO (time to diagnosis)
Nagaratnam et al, 2020 [52]	Observational study (retrospective)	1 patient	Royal Berkshire Hospital (United Kingdom)	PO (patient care and clinical outcomes)
Natarajan et al, 2019 [53]	Observational study (prospective)	213 patients	Dispensaries under Municipal Corporation of Greater Mumbai (India)	AP (sensitivity, specificity)
Nicolae et al, 2020 [54]	RCT	41 patients	Sunnybrook Odette Cancer Centre (Canada)	AP (day 30 dosimetry) CO (planning time)
Park et al, 2019 [55]	Experimental study (within subjects)	8 clinicians	Stanford University Medical Center (United States)	CO (specificity, sensitivity, accuracy interrater agreement, time to diagnosis)
Romero-Brufau et al, 2020 [56]	Pre-post survey	81 clinical staff	3 primary-care clinics in Southwest Wisconsin (United States)	CO (attitudes about AI^o^ in the workplace)
Rostill et al, 2018 [57]	RCT	204 patients, 204 caregivers	NHS, Surrey and Hampshire (United Kingdom)	CO (system evaluations) PO (early clinical interventions, patient evaluations)
Segal et al, 2014 [58]	Observational study (prospective)	16 pediatric neurologists	Boston Children’s Hospital (United States)	CO (diagnostic errors, diagnosis relevance, number of workup items)
Segal et al, 2016 [59]	Observational study (prospective)	26 clinicians	Boston Children’s Hospital (United States)	CO (diagnostic errors)
Segal et al, 2017 [60]	Structured interviews	10 medical specialists	Geisinger Health System and Intermountain Healthcare (United States)	CO (system perceptions)
Segal et al, 2019 [61]	Observational study (prospective)	3160 patients (315 prescription alerts)	Sheba Medical Center (Israel)	AP (accuracy, clinical validity, and usefulness) PO (changes in medical orders)
Shimabukuro et al, 2017 [62]	RCT	142 patients	University of California San Francisco Medical Center (United States)	PO (LOS, in-hospital mortality)
Sim et al, 2020 [63]	Observational study (prospective)	12 radiologists	4 medical centers (United States and South Korea)	AP (sensitivity, FPPI^p^) CO (sensitivity, FPPI, decision change)
Steiner et al, 2018 [64]	Experimental study (within subjects)	6 anatomic pathologists	NR	CO (sensitivity, average review per image, interpretation difficulty)
Su et al, 2020 [65]	RCT	623 patients, 6 endoscopists	Qilu Hospital of Shandong University (China)	CO (ADR, PDR, number of adenomas and polyps, withdrawal time, adequate bowel preparation rate)
Titano et al, 2018 [66]	RCT	2 radiologists	NR	CO (time to diagnosis, queue of urgent cases)
Vandenberghe et al, 2017 [67]	Observational study (prospective)	1 pathologist and 2 HER2 raters	NR	CO (decision concordance, decision modification)
Voerman et al, 2019 [68]	Before-after study	NR	Five Rivers Medical Center, Pocahontas (United States)	CE^q^ (average total costs per patient) PO (numbers of patients with clostridium difficile and antibiotic-resistant infections, LOS, antibiotic use)
Wang et al, 2019 [69]	RCT	1058 patients, 8 physicians	Sichuan Provincial People’s Hospital (China)	CO (ADR, PDR, number of adenomas per patient)
Wang et al, 2019 [70]	RCT	75 patients	4 primary care clinics affiliated with Brigham and Women’s Hospital (United States)	CO (anticoagulation prescriptions)
Wang et al, 2020 [71]	RCT	962 patients	Caotang branch hospital of Sichuan Provincial People’s Hospital (China)	CO (ADR, PDR, number of adenomas and polyps per colonoscopy)
Wijnberge et al, 2020 [72]	RCT	68 patients	Amsterdam UMC (Netherlands)	PO (median time-weighted average of hypotension, median time of hypotension, treatment, time to intervention, adverse events)
Wu et al, 2019 [73]	Observational study (prospective)	3600 residents	3 ophthalmologists, community healthcare centers (China)	AP (AUC) CO (ophthalmologist-to-population service ratio)
Wu et al, 2019 [74]	RCT	303 patients, 6 endoscopists	Renmin hospital of Wuhan University (China)	AP (accuracy, completeness of photo documentation) CO (blind spot rate, number of ignored patients, inspection time) PO (adverse events)
Yoo et al, 2018 [75]	Observational study (prospective)	50 patients, 1 radiologist	NR (Korea)	AP (sensitivity, specificity, PPV, NPV, accuracy) CO (sensitivity, specificity, PPV, NPV, accuracy)

^a^AP: application performance.

^b^CO: clinician outcomes.

^c^CT: computed tomography.

^d^AUC: area under the curve.

^e^RCT: randomized controlled trial.

^f^PO: patient outcomes.

^g^ICU: intensive care unit.

^h^LOS: length of stay.

^i^NR: not reported.

^j^PPV: positive-predictive value.

^k^NPV: negative-predictive value.

^l^ADR: adenoma detection rate.

^m^PDR: polyp detection rate.

^n^ACSC: ambulatory care sensitive admissions.

^o^AI: artificial intelligence.

^p^FFPI: false-positive per image.

^q^CE: cost-effectiveness.

### Quality Assessment

Considering the heterogeneity of study types included in the review, we only assessed the risk of bias of 13 RCTs using the Cochrane Collaboration Risk of Bias tool (Multimedia Appendix 2) [76]. Overall, the total score of the RCTs ranged from 0 [57] to 6 [44,65,69], with a mean value of 3.84. Specifically, eight studies reported random sequence generation [33,44, 62,65,69,71,72], and three studies explicitly stated that the allocation was concealed [62,65,72]. Only two studies were double blinded [66,71]. Blinding of participants was unsuccessful in two studies [62,72] and was unclear in six studies [44,46,54,57,69,70]. Blinding of outcome assessment was unsuccessful in seven studies [33,46,62,65,69,70,74] and was unclear in one study [57]. Three studies did not clearly state whether they had complete data for the enrolled participants [57,66,77]. All of the 13 studies had a low risk of selective reporting bias. Other potential sources of bias included a small sample size [62,70,72], a short study period [62], and a lack of detailed information regarding RCTs and follow-ups [57,66].

### AI Application Characteristics

Among the 51 studies, two did not disclose any information regarding the AI techniques used. Among the remaining 49 studies, the most popular ML technique was neural networks (n=22), followed by random forests (n=3), Bayesian pattern matching (n=3), support vector machine (n=2), decision tree (n=2), and deep reinforcement learning (n=2). We also found that the included AI applications mainly provided decision support in the following four categories of clinical tasks: disease screening or triage (n=16), disease diagnosis (n=16), risk analysis (n=14), and treatment (n=7). Further, AI applications in 46 (94%) studies targeted one or more specific diseases and conditions. The most prevalent diseases and conditions were sepsis (n=6), breast cancer (n=5), diabetic retinopathy (n=4), polyp and adenoma (n=4), cataracts (n=2), and stroke (n=2). Details of AI application characteristics are provided in Multimedia Appendix 3.

### Evaluation Outcomes

We categorized the evaluation outcomes in our review studies into the following four types: performance of AI applications, clinician outcomes, patient outcomes, and cost-effectiveness, as can be seen in Table 1 and Multimedia Appendix 4.

#### Performance of AI Applications

Twenty-six studies evaluated the performance of AI applications in real-life clinical settings [25,27,29-32,35,36,38-43,45,47, 48,51,52,54,61,63,73-75,78]. Commonly used performance metrics included accuracy, area under the curve (AUC), specificity, sensitivity, positive-predictive value (PPV), and negative-predictive value (NPV). Of these, 24 studies reported acceptable and satisfactory performance of AI applications in practice. For example, one study [25] conducted a pivotal trial of the IDx-DR diagnostic system (IDx, LLC) to detect diabetic retinopathy in 10 primary clinic offices in the United States. They reported that IDx-DR had a sensitivity of 87.2%, a specificity of 90.7%, and an imageability rate of 96.1%, exceeding prespecified endpoints. Based on the results, IDx-DR became the first Food and Drug Administration (FDA)–authorized AI diagnostic system, with the potential to improve early detection of diabetic retinopathy and prevent vision loss in thousands of patients with diabetes.

On the contrary, two studies found that AI applications failed to outperform health care providers and needed further improvement [40,44]. In particular, one RCT [44] examined the performance of CC-Cruiser, an AI-based platform for childhood cataracts, in five ophthalmic clinics in China. The authors found that CC-Cruiser had considerably lower accuracy, PPV, and NPV than senior consultants in diagnosing childhood congenital cataracts and making treatment decisions. Another study [40] evaluated the performance of an AI-based diabetic retinopathy grading system in a primary care office in Australia and found that the AI system had a high false-positive rate with a PPV of 12%. Specifically, of the 193 patients who consented to the study, the AI system identified 17 patients with severe diabetic retinopathy that required referral. However, only two patients were correctly identified, and the remaining 15 patients were false positives.

#### Clinician Outcomes

Thirty-three studies examined the effect of AI applications on clinician outcomes, that is, clinician decision making, clinician workflow and efficiency, and clinician evaluations and acceptance of AI applications [26,27,29,31-33,37,38, 42-47,50,51,54-60,64,65,67,69-71,73-75].

AI applications have the potential to provide clinical decision support. From our review, 16 studies demonstrated that AI applications could enhance clinical decision-making capacity [31-33,45-47,50,55,58,59,63,64,67,69,71,74,75]. For example, Brennan et al [32] found that clinicians gained knowledge after interacting with MySurgery, an algorithm for preoperative risk assessments, and improved their risk assessment performance as a result. On the contrary, two studies did not find any evidence for enhanced decision-making [26,42]. One possible explanation is that AI may provide misleading recommendations, offsetting the benefits of AI. Specifically, Kiani et al [42] evaluated the effect of a DL-based system for live cancer classification on the diagnostic performance of 11 pathologists and found that AI use did not greatly improve the diagnostic accuracy. They further noted that AI improved accuracy when it provided correct predictions and harmed accuracy when it provided wrong predictions. Aoki et al [26] examined the impact of a DL-based system for mucosal break detection on endoscopists in reading small bowel capsule endoscopy. They found that the system failed to improve the mucosal break detection performance of endoscopists, particularly trainees.

Seven studies were aimed at clinician workflow and efficiency [26,27,44,51,54,66,73]. Of these, six studies found that AI accelerated the time needed for clinical tasks and improved the existing workflow [26,27,44,51,54,66]. For example, Titano et al [66] found that a DL-based cranial image triage algorithm processed and interpreted images 150 times faster than human radiologists (1.2 seconds vs 177 seconds) and appropriately escalated urgent cases, enhancing the triage of cases in the radiology workflow. The only exception is the work of Wu et al [74], which assessed the quality improvement system WISENSE for blind-spot monitoring and procedure timing during esophagogastroduodenoscopy. This study found that WISENSE helped endoscopists monitor and control their time on each procedure and increased inspection time as a result.

Finally, clinician perceptions and acceptance of AI applications were examined in seven studies [32,37,38,43,56,57,60]. Particularly, five out of the seven studies reported overall positive perceptions of AI applications [32,38,43,57,60]. For example, Brennan et al [32] asked 20 surgical intensivists to use and evaluate MySurgeryRisk for preoperative risk prediction in a simulated clinical workflow. Most respondents indicated that MySurgeryRisk was useful and easy to use and believed that it would be helpful for decision making. On the other hand, the remaining two studies reported mixed or even negative evaluations of AI [37,56]. Specifically, Ginestra et al [37] assessed physician evaluations of an ML-based sepsis prediction system in a tertiary teaching hospital and found that only 16% of health care providers perceived system-generated sepsis alerts to be helpful. The negative evaluations could be attributed to providers’ low confidence in alerts, low algorithm transparency, and a lack of established actions after alerts. Romero-Brufau et al [56] reported survey results from implementing an AI-based clinical decision support system in a regional health system practice and found that only 14% of clinical staff were willing to recommend the system. Staff feedback revealed that some system-recommended interventions were inadequate and inappropriate.

#### Patient Outcomes

Fourteen studies reported patient outcomes [28,34, 36,41,44,48,49,52,57,61,62,68,72,74]. In 11 of the 14 studies, researchers examined the effect of AI on clinical processes and outcomes, such as hospital length of stay, in-hospital mortality, intensive care unit (ICU) transfer, readmission, and time to intervention [28,34,36, 48,49,52,57,61,62,68,72,74]. The results were inconsistent. Most studies reported improved clinical outcomes (n=8) [36,48,52,57,61,62,68,72,74]. For example, one RCT [62] implemented and assessed an ML-based severe sepsis prediction algorithm (Dascena) in two ICUs at the University of California San Francisco Medical Center. They found that the algorithm implementation greatly decreased the hospital length of stay from 13.0 days to 10.3 days and decreased the in-hospital mortality rate from 21.3% to 8.96%. However, three of the studies did not find evidence for improved clinical outcomes, indicating the limited applicability of the algorithms in their current form [28,34,36]. In particular, Bailey et al [28] examined the effect of an ML-based algorithm that generated real-time alerts for clinical deterioration in hospitalized patients. They found that providing alerts alone could not reduce the hospital length of stay and the in-hospital mortality. Connell et al [34] examined the effect of a novel digitally enabled care pathway for acute kidney injury management and found no step changes in the renal recovery rate and other secondary clinical outcomes following the intervention. Giannini et al [36] developed and implemented a sepsis prediction algorithm in a tertiary teaching hospital system. The results showed that the algorithm-generated alerts had a limited impact on clinical processes and could not reduce mortality, discharge dispositions, or transfer to the ICU. Future algorithm optimization is thus needed.

Three studies examined how patients evaluated AI applications, and all of them reported positive results [41,44,57]. Keel et al [41] evaluated patient acceptability of an AI-based diabetic retinopathy screening tool in an endocrinology outpatient setting. They found that 96% (92/96) of the screened patients were satisfied with the AI tool and 78% (43/55) of the patients in the follow-up survey preferred AI screening over manual screening, suggesting that the AI tool was well-accepted by patients. Lin et al [44] assessed patient satisfaction with CC-Cruiser for childhood cataracts and found that patients were slightly more satisfied with CC-Cruiser in comparison with senior consultants. One explanation is that childhood cataracts may cause irreversible vision impairment and even blindness without early intervention. Therefore, parents of patients appreciated the faster diagnosis of CC-Cruiser. Rostill et al [57] assessed an Internet of Things (IoT) system for dementia care and found that dementia patients trusted the system and would like to recommend it.

#### Cost-Effectiveness

The economic impact of AI implementation in clinical practice was addressed in only one study [68]. This study reported that the implementation of an ML-based system for antibiotic stewardship reduced costs by US $25,611 for sepsis and US $3630 for lower respiratory tract infections compared with usual care.

## Discussion

### Principal Findings

AI applications have huge potential to augment clinician decision making, improve clinical care processes and patient outcomes, and reduce health care costs. Our review seeks to identify and summarize the existing studies on AI applications that have been implemented in real-life clinical practice. It yields the following interesting findings.

First, we note that the number of included studies was surprisingly small considering the tremendous number of studies on health care AI. In particular, most of the health care AI studies were proof-of-concept studies that focused on AI algorithm development and validation using retrospective clinical data sets. In contrast, only a handful of studies implemented and evaluated AI in a clinical environment. To ensure safe adoption, however, an AI application should provide solid scientific evidence for its effectiveness relative to the standard of care. Therefore, we urge the health care AI research community to work closely with health care providers and institutions to demonstrate the potential of AI in real-life clinical settings.

Second, more than two-thirds of the included articles were from developed economies, of which more than half were from the United States, suggesting that developed countries are at the forefront of health care AI development and deployment. This is consistent with the fact that top health AI companies and start-ups (eg, Google Health, IBM Watson Health, and Babylon Health) are mainly located in the United States and Europe. This finding should be interpreted with caution because we excluded non-English–written articles, even though our search had identified 890 non-English publications. We did not include these non-English articles because it is difficult to conduct an unbiased analysis owing to translation difficulty and variation. The imbalanced distribution of articles by country or economic development status could be attributed to the fact that researchers from low-income countries have a very low publication rate.

However, it is worth noting that 8 (16%) of our articles were from China, suggesting that China has been extensively applying health care AI and conducting health care AI research. Indeed, hospitals, technology companies, and the Chinese government have been driving clinical AI deployment with the aim to alleviate doctor shortages, relieve medical resource inequality, and reduce health care costs [79-82], and Chinese researchers have acquired the capability to publish in international English journals.

Third, the quality of research on clinical AI evaluation needs to be improved in the future. Our review revealed that only 13 (26%) studies were RCTs and most of them suffered from moderate to high risk of bias. Eight studies were experimental studies, and all of them adopted a cross-over design or within-subjects design and were hence susceptible to confounding effects. With respect to sample information, only 8 (16%) studies provided information on both patients and health care providers, and 14 (28%) studies used a sample size smaller than 20 (Table 1), limiting the generalizability of their results. Regarding the evaluation design, one-third of the studies (n=17, 33%) did not include a comparison group (Multimedia Appendix 4), limiting the ability to identify the added value of AI applications compared with the current best practice. Given that health care providers may hold different perceptions toward different AI systems of varying performance and reliability, it would be helpful if the studies provide a transparent description of the AI system’s architecture, accuracy or reliability performance, and possible risks. Unfortunately, in our review, 21 studies did not provide adequate information about the architecture of the AI applications [25,29,32,34, 37,44,46,50,52,54,56-62,65,68,70,75] and 22 studies did not reveal the performance and possible risks of AI under evaluation [26,29,34,37,39,46,48-50,52,54,56-62,64,65,68,69]. Further, considering that some self-evolving adaptive clinical AI applications continuously incorporate the latest clinical practice data and published evidence, it is important to undertake periodic monitoring and recalibration of AI applications to ensure that they are working as expected. Finally, we found that more than half of the studies (n=29, 57%) investigated only one aspect of evaluation outcome (Multimedia Appendix 4). We encourage future research to conduct a more comprehensive assessment of the quality of clinical AI applications as well as their impacts on clinicians, patients, and health care institutions. This will facilitate the comparison and selection of alternative AI solutions in the same clinical domain.

Fourth, our analysis indicated that AI applications could provide effective decision support, albeit in certain contexts. For instance, the augmenting role of AI in clinical decision-making capacity can be affected by the level of expertise. In particular, two studies suggested that junior physicians were more likely to benefit from AI than senior physicians because they had a higher tendency to reconsider and modify their clinical decisions when encountering disconfirming AI suggestions [38,47]. However, it is worth noting that AI can be misleading sometimes. For example, one study from our review speculated that trainee endoscopists may feel confused about false-positive results from an AI screening tool owing to limited reading experience and, as a result, ignore AI-marked lesions of small-bowel mucosal breaks [26]. It is therefore important for future research to examine under what circumstances physicians could benefit more from AI applications. However, we are sanguine that when AI technology is sufficiently mature and accurate to become the evidence-based best practice, its use would become part of routine clinical care in the future.

With respect to AI acceptance, we observed that health care providers expressed negative feelings toward AI in two studies [37,56], indicating that barriers existed in the incorporation of AI into the routine workflow. However, an elaboration of AI implementation barriers will be lengthy and is beyond the scope of this work, and we refer interested readers to the reports by Kelly et al [8], Ngiam and Khor [83], Lysaght et al [84], Shaw et al [10], and Yu and Kohane [12] for more details.

Fifth, for most of the included studies on patient outcomes, we found that they did not examine the clinical processes and interventions in detail. However, AI applications without appropriate and useful interventions may be ineffective at improving patient outcomes. For example, Bailey et al [28] found that simply notifying the nursing staff of clinical deterioration risks was not able to improve the outcomes of high-risk patients. Effective patient-specific interventions are needed. Therefore, future research may design and evaluate patient-directed interventions to enhance the clinical effectiveness of AI applications.

Moreover, three of the included studies suggested that patients and their families were highly satisfied with health care AI owing to its convenience and efficiency [41,44,57]. However, this may not always be the case. Prior research has shown that patients preferred to receive primary care from a human provider than AI even if the care from the health provider entailed a higher misdiagnosis risk [85]. The reason is that they perceived AI to be less capable in considering their unique circumstances. Additionally, patients may disparage physicians aided by a clinical decision support system and perceive them as less capable and professional than their unaided counterparts [86]. Further studies to explore the possible patient concerns and resistance toward health AI applications should be considered.

Finally, according to an Accenture survey, more than half of health care institutions are optimistic that AI will reduce costs and improve revenue despite the high initial costs associated with AI implementation [87]. However, only one included study documented the economic outcomes of AI implementation. This highlights the need to conduct more cost-effectiveness analyses of AI applications in clinical practice.

### Limitations

This review has several limitations. First, we only included peer-reviewed English-written journal articles. It is plausible that some relevant articles were written in other languages or published in conferences, workshops, and news reports. As noted earlier, this may partly explain the imbalanced country distribution of the reviewed articles. Moreover, we did not include articles that were published before 2010 because AI only started to make in-roads in the clinical field in the last decade, as evident in our search results. Moreover, we only reviewed premium computer science conferences and journals without comprehensively examining engineering and computer science databases. This should be less of a concern here because we found that computer science conferences and journals mainly focus on the training and validation of novel AI algorithms without actual deployment. Still, future research can expand the search scope to gain deeper insights into state-of-the-art clinical AI algorithms.

Another concern is that some AI applications may have been implemented in real-world clinical practice without any openly accessible publications. For example, IDx-DR, the first FDA-approved AI system, has been implemented in more than 20 health care institutions such as University of Iowa Health Care [88]. However, our search only identified one related published result [25]. Clinical practitioners should take a more active role in reporting AI evaluation and use results in their daily practice in the future.

### Conclusions

AI applications have tremendous potential to improve patient outcomes and improve care processes. Based on the literature presented in this review, there is great interest to develop AI tools to support clinical workflows, with increasing high-quality evidence being generated. However, there is currently insufficient level 1 evidence to advocate the routine use of health care AI for decision support, hindering the growth of health care AI and presenting potential risks to patient safety. We thus conclude that it is important to conduct robust RCTs to benchmark AI-aided care processes and outcomes to the current best practice. A rigorous, robust, and comprehensive evaluation of health care AI will help move from theory to clinical practice. 

## Appendix

Multimedia Appendix 1 Search strategy.

Multimedia Appendix 2 Quality assessments of randomized controlled trials based on the Cochrane Collaboration Risk of Bias Tool.

Multimedia Appendix 3 Artificial intelligence application characteristics.

Multimedia Appendix 4 Evaluation outcomes and main results of the included studies.

## Abbreviations

AI: artificial intelligence
DL: deep learning
FDA: Food and Drug Administration
ICU: intensive care unit
ML: machine learning
NLP: natural language processing
NPV: negative-predictive value
PPV: positive-predictive value
RCT: randomized controlled trial
