# Supplementary material for: Role of Artificial Intelligence Applications in Real-Life Clinical Practice: Systematic Review
Source: J Med Internet Res. 2021 Apr 22;23(4):e25759. doi: 10.2196/25759 (PMC8103304; doi:10.2196/25759)
Supplement: Multimedia Appendix 1 [file jmir_v23i4e25759_app1.pdf]

Multimedia Appendix 1. Search strategy

Keywords

(1) Artificial intelligence (combine using OR)

- artificial intelligence
- machine learning
- deep learning

(2) Clinical implementation (combine using OR)

- clinical
- health
- healthcare
- medical
- implement
- implementation
- deploy
- deployment
- adoption

PubMed (n=6830)

Search query 1: ("artificial intelligence"[Title/Abstract] OR "machine learning"[Title/Abstract] OR "deep learning"[Title/Abstract]) **AND** ("clinical"[Title/Abstract] OR "health"[Title/Abstract] OR "healthcare"[Title/Abstract] OR "medical"[Title/Abstract] OR "implement"[Title/Abstract] OR "implementation"[Title/Abstract] OR "deploy"[Title/Abstract] OR "deployment"[Title/Abstract] OR "adoption"[Title/Abstract])
Filters: Full text, Journal Article, Humans, English, from 2010/1/1 - 2020/5/31
(n=8144)
Search query 2: ("artificial intelligence"[Title/Abstract] OR "machine learning"[Title/Abstract] OR "deep learning"[Title/Abstract]) **AND** ("clinical"[Title/Abstract] OR "health"[Title/Abstract] OR "healthcare"[Title/Abstract] OR "medical"[Title/Abstract] OR "implement"[Title/Abstract] OR "implementation"[Title/Abstract] OR "deploy"[Title/Abstract] OR "deployment"[Title/Abstract] OR "adoption"[Title/Abstract])
Filters: Full text, Bibliography, Biography, Books and Documents, Clinical Conference, Clinical Trial Protocol, Comment, Electronic Supplementary Materials, English Abstract, Meta-Analysis, Newspaper Article, Review, Systematic Review, Humans, English, from 2010/1/1 - 2020/5/31
(n=1347)
Search query 1 – search query 2

CINAHL (n=839)

TI (("artificial intelligence" OR "machine learning" OR "deep learning") AND ("clinical" OR "health" OR “healthcare" OR "medical" OR "implement" OR "implementation" OR “deploy" OR "deployment" OR "adoption")) OR AB (("artificial intelligence" OR "machine learning" OR "deep learning") AND ("clinical" OR "health" OR “healthcare" OR "medical" OR "implement" OR "implementation" OR “deploy" OR "deployment" OR "adoption")) OR MW (("artificial intelligence" OR "machine learning" OR "deep learning") AND ("clinical" OR "health" OR “healthcare" OR "medical" OR "implement" OR "implementation" OR “deploy" OR "deployment" OR "adoption"))
Limiters - Full Text; Published Date: 20100101-20200531

Expanders - Apply equivalent subjects
Narrow by Language: - english
Search modes - Boolean/Phrase

Embase (n=9124)
(('artificial intelligence':ti,ab,kw OR 'machine learning':ti,ab,kw OR 'deep learning':ti,ab,kw) AND ('clinical':ti,ab,kw OR 'health':ti,ab,kw OR 'healthcare':ti,ab,kw OR 'medical':ti,ab,kw OR 'implement':ti,ab,kw OR 'implementation':ti,ab,kw OR 'deploy':ti,ab,kw OR 'deployment':ti,ab,kw OR adoption:ti,ab,kw) AND [humans]/lim AND [1-1-2010]/sd NOT [1-6-2020]/sd AND [article]/lim AND [english]/lim) AND 'article'/it

Cochrane Central (n=1152)
"artificial intelligence" OR "machine learning" OR "deep learning" in Title Abstract Keyword
Added Date: 20100101-20200531

List of premium computer science journals and conferences

- Artificial Intelligence
- IEEE Transactions on Pattern Analysis and Machine Intelligence
- IEEE Transactions on Image Processing
- IEEE Transactions on Neural Networks
- International Journal of Computer Vision
- Journal of AI Research
- Journal of Machine Learning Research
- Neural Computation
- Machine Learning
- American Association for AI National Conference (AAAI)
- Annual Meeting of the Association of Computational Linguistics (ACL)
- Annual Conference on Neural Information Processing Systems (NeurIPS)
- Conference on Uncertainty in AI (UAI)
- Empirical Methods in Natural Language Processing (EMNLP)
- Knowledge Discovery and Data Mining (KDD)
- IEEE Conference on Computer Vision and Pattern Recognition (CVPR)
- International Conference on Computer Vision (ICCV)
- International Conference on Machine Learning (ICML)
- International Conference on the Principles of Knowledge Representation and Reasoning (KR)
- International Joint Conference on Artificial Intelligence (IJCAI)
- Robotics: Science & Systems (RSS)
