# Supplementary material for: Role of Artificial Intelligence Applications in Real-Life Clinical Practice: Systematic Review
Source: J Med Internet Res. 2021 Apr 22;23(4):e25759. doi: 10.2196/25759 (PMC8103304; doi:10.2196/25759)
Supplement: Multimedia Appendix 3 [file jmir_v23i4e25759_app3.pdf]

Multimedia Appendix 3. AI application characteristics

Author, year	AI applications	AI techniques	Clinical tasks	Disease domains and conditions
Abràmoff et al, 2018 [25]	IDx-DR diagnostic system (IDx LLC, Coralville, IA) for automatic detection of diabetic retinopathy and diabetic macular edema based on retinal images	ML (CNN[a])	Disease diagnosis	Diabetic retinopathy and diabetic macular edema
Aoki et al, 2020 [26]	A deep learning-based system that automatically detects mucosal breaks based on small-bowel capsule endoscopy images	ML (CNN)	Disease screening	Small-bowel diseases
Arbabshirani et al, 2018 [27]	A deep learning algorithm for intracranial hemorrhage diagnosis based on computed tomography scans of the head	ML (CNN)	Disease diagnosis	Intracranial hemorrhage
Bailey et al, 2013 [28]	A real-time algorithm for clinical deterioration for patients in general medical wards based on vital sign measures	ML (DT[a], LR[a])	Risk analysis	Sepsis
Barinov et al, 2019 [29]	An AI-based decision support system for breast cancer diagnosis based on ultrasound images	ML (NR[b])	Disease diagnosis	Breast cancer
Beaudoin et al, 2016 [30]	A clinical decision support system for antimicrobial stewardship based on patient clinical data	ML (rule induction, instance-based learning)	Treatment	General: antimicrobial prescriptions
Bien et al, 2018 [31]	MRNet, a diagnostic algorithm based on knee magnetic resonance imaging	ML (CNN)	Disease diagnosis	Anterior cruciate ligament tears; meniscal tears
Brennan et al, 2019 [32]	MySurgeryRisk, an algorithm for automated preoperative risk assessment based on the EHR data	ML (RF[a])	Risk analysis	Surgery-related risk
Chen et al, 2020 [33]	ENDOANGEL (previous WISENSE AI system), a real-time AI system that monitors blind spots during EGD	ML (CNN, DRL[a])	Disease screening	Esophageal, gastric, and duodenal diseases
Connell et al, 2019 [34]	Streams (DeepMind Technologies Ltd, London, UK), a digitally-enabled care pathway for acute kidney injury management that incorporated in a mobile application	ML (NR)	Risk analysis	Acute kidney injury
Eshel et al, 2017 [35]	Parasight Platform (SightDX, Tel Aviv), an automated system that provides malaria diagnosis, species identification, and parasite quantification based on blood samples	ML (SVM[a])	Disease diagnosis	Malaria
Giannini et al, 2019 [36]	Early Warning System 2.0 (EWS 2.0), an ML-based tool that provides severe sepsis and sepsis shock prediction based on the clinical data from EHR	ML (RF)	Risk analysis	Sepsis
Ginestra et al, 2019 [37]	An early warning system that provides severe sepsis and sepsis shock prediction based on the clinical data from EHR	ML (NR)	Risk analysis	Sepsis

Gómez-Vallejo et al, 2016 [38]	InNoCBR, a case-based reasoning system for automatic healthcare-associated infection surveillance and diagnosis	ML (NB[a], PART[a], DT)	Risk analysis	Healthcare-associated infections
Grunwald et al, 2016 [39]	Electronic Alberta Stroke Program Early CT (e-ASPECT) (Brainomix Limited, Summertown, Oxford, UK), a tool for early detection of ischemic damage based on CT scans	ML (RF)	Triage; Treatment	Stroke
Kanagasingam et al, 2018 [40]	An AI-based grading system for diabetic retinopathy based on retinal images	ML (CNN)	Disease screening	Diabetic retinopathy
Keel et al, 2018 [41]	A deep learning algorithm for referrable diabetic retinopathy screening based on retinal images	ML (CNN)	Disease screening	Diabetic retinopathy
Kiani et al, 2020 [42]	A DL-based system for histopathologic classification of liver cancer (i.e., hepatocellular carcinoma, cholangiocarcinoma) based on whole-slide images	ML (CNN)	Disease diagnosis	Liver cancer
Lagani et al, 2015 [43]	An AI-based system that predicts the long-term risk of diabetes-related complications based on individual patient profiles	ML (cox model, AFT[a] model, RSF[a], SCVR[a])	Risk analysis	Diabetes
Lin et al, 2019 [44]	CC-Cruiser platform, an AI-based online platform for congenital cataract based on eye images	ML (CNN)	Disease diagnosis	Childhood cataracts
Lindsey et al, 2018 [45]	A deep learning algorithm to detect and localize fractures in radiographs	ML (CNN)	Disease diagnosis	Fracture detection
Liu et al, 2020 [46]	An algorithm for polyp and adenoma detection in colonoscopy (Henan Xuanweitang Medical Information Technology Co., LTD., Zhengzhou, Henan, China)	ML (CNN)	Disease screening	Polyp and adenoma
Mango et al, 2020 [47]	Koios DS for Breast, an AI-based system that provides decision support for breast ultrasound lesion assessment (Koios Medical, NY, US)	ML (NR)	Disease diagnosis	Breast cancer
Martin et al, 2012 [48]	Patient Journey Record system, a system that detects adverse changes in patient biopsychosocial trajectories	ML (rule-based algorithms)	Risk analysis	General
McCoy and Das, 2017 [49]	InSight (Dascena, Hayward, California), a sepsis prediction algorithm based on EHR data	ML (NR)	Risk analysis	Sepsis
McNamara et al, 2019 [50]	Watson for Oncology with Cota RWE platform (IBM Watson Health, Cambridge, MA), a clinical decision support system that provides treatment recommendations for patients with cancer	ML (NR)	Treatment	Breast cancer
Mori et al, 2018 [51]	An AI-based CAD system in assessing diminutive polyps during colonoscopy	ML (SVM)	Disease screening	Diminutive polyps
Nagaratnam et al, 2020 [52]	e-Stroke Suite (Brainomix Limited, Summertown, Oxford, UK)	ML (DL)	Treatment	Hyperacute stroke
Natarajan et al, 2019 [53]	Medios AI (Remidio Innovative Solutions Pvt Ltd, Karnataka, India), a mobile-based automated system for	ML (CNN)	Disease screening	Diabetic retinopathy

	diabetic retinopathy screening based on retinal images			
Nicolae et al, 2020 [54]	An ML-based prostate implant planning algorithm	ML (kNN[a])	Treatment	Prostate cancer
Park et al, 2019 [55]	HeadXNet Model, a segmentation model that predicts intracranial aneurysms based on head computed tomographic angiography imaging	ML (CNN)	Disease diagnosis	Cerebral aneurysms
Romero-Brufau et al, 2020 [56]	A commercial AI-based clinical decision support tool to improve glycemic control in patients with diabetes	NR	Risk analysis	Diabetes
Rostill et al, 2018 [57]	Technology integrated health management, an IoT system for dementia care	ML (Markov chain model)	Risk analysis	Dementia
Segal et al, 2014 [58]	SimulConsult diagnostic decision support system (SimulConsult, Chestnut Hill, MA), a system that informs diagnostic assessments based on the temporal pattern of clinical findings in each disease	ML (Bayesian pattern matching)	Disease diagnosis	Neurological disorders
Segal et al, 2016 [59]	SimulConsult diagnostic decision support system	ML (Bayesian pattern matching)	Disease diagnosis	Pediatric rheumatologic disorders
Segal et al, 2017 [60]	SimulConsult diagnostic decision support system	ML (Bayesian pattern matching)	Disease diagnosis	Child neurology diseases
Segal et al, 2019 [61]	MedAware, an ML-based system for identification and prevention of prescription error	ML (NR)	Treatment	General: prescriptions
Shimabukuro et al, 2017 [62]	A commercial sepsis prediction algorithm (Dascena, Hayward, California)	ML (NR)	Risk analysis	Sepsis
Sim et al, 2020 [63]	Samsung Auto Lung Nodule (ALAND) (Samsung Electronics, Suwon, South Korea), an ML-based software that detects malignant lung nodules on chest radiographs	ML (CNN)	Disease screening	Malignant pulmonary nodules
Steiner et al, 2018 [64]	A deep learning algorithm that reviews lymph nodes for metastatic breast cancer diagnosis	ML (CNN)	Disease screening	Breast cancer metastasis
Su et al, 2020 [65]	An automated system for polyp and adenoma detection in colonoscopy (Qingdao Medicon Digital Engineering Co Ltd, Shandong, China)	ML (CNN)	Disease screening	Colorectal cancer
Titano et al, 2018 [66]	An algorithm for automated surveillance and triage of cranial images	ML (CNN)	Disease diagnosis; Triage	Acute neurologic events
Vandenberghe et al, 2017 [67]	An algorithm that recognizes cancer cell types and predicts HER2 status in breast cancer based on tumor resection samples (AstraZeneca, Cambridge, UK)	ML (CNN)	Disease diagnosis	Breast cancer
Voerman et al, 2019 [68]	A procalcitonin-guided decision algorithm for antibiotic stewardship	ML (NR)	Treatment	Sepsis and lower respiratory tract infections

Wang et al, 2019 [69]	A real-time automatic polyp and adenoma detection system in colonoscopy (Shanghai Wision AI, Shanghai, China)	ML (CNN)	Disease screening	Polyp and colorectal adenoma
Wang et al, 2019 [70]	An algorithm that identifies patients with atrial fibrillation and recommends anticoagulation usage based on EHR data	ML (NR)	Risk analysis	Atrial fibrillation
Wang et al, 2020 [71]	EndoScreener, a polyp and adenoma detection system in colonoscopy (Shanghai Wision AI, Shanghai, China)	ML (deep learning)	Disease screening	Polyp and adenoma
Wijnberge et al, 2020 [72]	An early warning system for intraoperative hypotension based on hemodynamic variables (Edwards Lifesciences, Irvine, California)	ML	Risk analysis	Hypertension
Wu et al, 2019 [73]	Cataract AI agent (Beijing Tulip Partners Technology, Beijing, China), a platform for collaborative management of cataracts based on retinal images	ML (CNN)	Disease screening	Cataract
Wu et al, 2019 [74]	WISENSE, a real-time AI system that monitors blind spots during EGD and facilitates the diagnosis of upper gastrointestinal lesions	Machine learning (CNN, DRL)	Disease screening	Gastric cancer
Yoo et al, 2018 [75]	A real-time computer-aided system for thyroid nodule diagnosis based on ultrasonography images (S-Detect for Thyroid; Samsung Medison Co., Ltd)	NR	Disease diagnosis	Thyroid cancer

[a]CNN: convolutional neural networks; DT: decision tree; LR: logistic regression; RF: random forests; DRL: deep reinforcement learning; SVM: support vector machine; NB: naïve Bayes; PART: partial decision tree algorithm; AFT model: accelerated failure time model; RSF: random survival forest; SCVR: support vector machine censored regression; kNN: k-nearest neighbor.

[b]NR: not reported.
