# Supplementary material for: Role of Artificial Intelligence Applications in Real-Life Clinical Practice: Systematic Review
Source: J Med Internet Res. 2021 Apr 22;23(4):e25759. doi: 10.2196/25759 (PMC8103304; doi:10.2196/25759)
Supplement: Multimedia Appendix 4 [file jmir_v23i4e25759_app4.pdf]

Multimedia Appendix 4. Evaluation outcomes and main results of the included studies

Author (year)	Implementation of AI systems	Comparison group(s) (if any)	Findings			
			Application performance (AP)	Clinician outcomes (CO)	Quality of care (QOC)	Economic impacts (EI)
Abràmoff et al, 2018 [25]	Conduct a trial of IDx-DR diagnostic system to detect diabetic retinopathy<br>Length: 7 months	N/A[a]<br>(set pre-defined primary endpoint goals)	Sensitivity: 87.2% (>85%)<br>Specificity: 90.7% (>82.5%)<br>Imageability rate: 96.1%	N/A	N/A	N/A
Aoki et al, 2020 [26]	Endoscopist readings after the first screening by the AI system for mucosal break detection<br>Task: 20 videos of small-bowel capsule endoscopy procedure	Endoscopist-alone readings	N/A	Reading time: I[b] (expert, 3.1 min; trainee, 5.2 min) vs. C[b] (expert, 12.2 min; trainee, 20.7 min), p<0.001<br>Detection rate of mucosal break: ns[c]	N/A	N/A
Arbabshirani et al, 2018 [27]	Re-prioritize head CT studies by implementing a DL model for intracranial hemorrhage (ICH) diagnosis<br>Length: 3 months	ICH detection in routine studies	AUC: 0.846<br>Accuracy: 84%<br>Sensitivity: 70%<br>Specificity: 87%	Time to diagnosis: I (19 min) vs. C (512 min), p<0.0001<br>94 cases were upgraded, and 5 new ICH cases were identified.	N/A	N/A
Bailey et al, 2013 [28]	Patients receive real-time alerts an AI sepsis prediction tool<br>Length: 4 years and 5 months	Patients without real-time alerts of the AI tool	N/A	N/A	ICU[d] transfer: patients flagged at high-risk were at higher risk (15.2% vs. 2.9%)<br>Hospital mortality: ns<br>LOS[d]: ns	N/A
Barinov et al, 2019 [29]	Sequential workflow<br>Task: 500 lesion cases	Independent workflow	AUC: 0.864 (>radiologist alone)	AUC: higher AUC in independent workflow<br>Kendall’s tau-b: 0.529-0.597	N/A	N/A
Beaudoin et al, 2016 [30]	Implement a system (baseline system with learning module) for antimicrobial stewardship<br>Length: 2 months	Baseline model (B), learning module for antimicrobial stewardship (L)	Trigger 43 recommendations out of 270 alerts (B: 38/240; L: 17/105)<br>Precision: 74% (B: 82%; L: 62%)	N/A	N/A	N/A

			Recall: 96% (B: 94%; L: 31%)<br>Accuracy: 79% (B: 85%; L: 51%)			
Bien et al, 2018 [31]	Clinical experts assisted by MRNet<br>Task: 60 exams	Clinical experts without assistance from MRNet	AUC (abnormality, ACL tears, meniscal tears): 0.937, 0.965, 0.937	Abnormality: ns<br>ACL tears: higher sensitivity (p=0.002) and specificity (p<0.001)<br>Meniscal tears: higher specificity (p=0.003)	N/A	N/A
Brennan et al, 2019 [32]	Physicians with assistance from MySurgeryRisk<br>Task: 150 patient cases	Physicians without assistance	AUC: 0.73-0.85 (higher than physicians' initial assessments, 0.47-0.69)	Decision changes: changed in 75% cases, change 8%-10% risk scores on average<br>AUC: increase by 5% in predicting cardiovascular complications (other complications: ns)<br>Usability: mixed	N/A	N/A
Chen et al, 2020 [33]	Patients undergo EGD assisted by ENDOANGEL<br>Task: 217 patients (lost to follow up not reported)	Patients undergo EGD without AI assistance<br>Task: 218 patients (lost to follow up not reported)	N/A	Lower blind spot rate in sedated C-EGD (I: 3.42%, C: 22.46%), unsedated U-TOE (I: 21.77%, C: 29.92%), unsedated C-EGD (I: 31.23%, C: 42.46%) (p<0.001)	N/A	N/A
Connell et al, 2019 [34]	Implementation of Streams (digitally enable care pathway) in one hospital	No Streams implemented in another hospital	N/A	N/A	Renal recovery rate: no compelling effects (ns)<br>Secondary clinical outcomes: no compelling effects (ns)<br>Care process: reduced unrecognized AKI cases (p<0.001), reduced time from ED registration to AKI recognition (p<0.001), reduced time to treatment of nephrotoxins (p=0.047).	N/A

					No significant differences in the release time of creatinine tests and time to treatment for patients with sepsis-related AKI and obstruction.	
Eshel et al, 2017 [35]	Microscopists use Parasight Platform for malaria diagnosis<br>Task: 205 samples in India (IN) 263 samples in Kenya (KE)	Microscopy<br>Rapid diagnostic test (RDT)<br>PCR	Sensitivity: 99% (IN), 99.3% (KE)<br>Specificity: 100% (IN), 98.9% (KE)<br>P. vivax/ P. falciparum. identification accuracy: 100%/ 100% (IN), 100%/ 96.1% (KE)<br>Device parasite count correlation: 0.84 (IN), 0.85 (KE)	N/A	N/A	N/A
Giannini et al, 2019 [36]	Implement Early Warning System 2.0 to predict sepsis<br>Silent period: 6 months<br>Alert period: 8 months	Pre-implementation period	Sensitivity: 26%<br>Specificity: 98%	N/A	Mortality: ns<br>Discharge disposition: ns<br>ICU transfer: ns<br>Reduced time-to-ICU transfer ($p<0.01$)<br>Clinical processes: limited changes	N/A
Ginestra et al, 2019 [37]	Implement an early warning system for severe sepsis prediction<br>Length: 6 weeks	N/A	N/A	Perceptions: 42% nurse and 16% providers perceived the alerts to be helpful. Nurses (13%) and providers (40%) differed in perceptions of sepsis presence at the time of alert. Few changed their perceptions (30% nurses and 9% providers).	N/A	N/A
Gómez-Vallejo et al, 2016 [38]	Deploy InNoCBR in a public hospital<br>Length: 10 months	N/A	Accuracy: 70.21%	System perceptions: the system is effective at decreasing infections and may		N/A

				reduce needed manpower by 70%.		
Grunwald et al, 2016 [39]	Implement e-ASPECT into an ambulance	N/A	E-ASPECTS results matched analysis of neuroradiologist.	N/A	N/A	N/A
Kanagasingam et al, 2018 [40]	Deploy an AI-based system for diabetic retinopathy in clinical practice<br>Length: 6 months	N/A	Sensitivity: 2 patients with severe disease were correctly identified<br>Specificity: 92%<br>PPV[d]: 12%<br>NPV[d]: 100%	N/A	N/A	N/A
Keel et al, 2018 [41]	Deploy a DL learning algorithm for referrable diabetic retinopathy<br>Length: 4 months	N/A	Sensitivity: 92.3%<br>Specificity: 93.7%<br>Mean assessment time: 6.9 min	N/A	Patient acceptability: 96% patients are satisfied and 78% preferred AI over manual approach.	N/A
Kiani et al, 2020 [42]	Pathologists assisted by a DL-based system for liver cancer classification<br>Task: 80 samples	Unassisted pathologists	Accuracy: 0.842	Accuracy: ns among 11 pathologists; AI improved accuracy among 9 pathologists with well-defined experience levels ($p = 0.045$); AI improved accuracy when it was correct ($p<0.001$) and decreased accuracy when it was wrong ($p<0.001$).	N/A	N/A
Lagani et al, 2015 [43]	An AI-based system that predicts the long-term risk of diabetes-related complications	N/A	Performance: comparable to UKPDS	Usability: overall positive feedback and criticisms for the system interface	N/A	N/A
Lin et al, 2019 [44]	Patients received diagnosis from CC-Cruiser platform<br>Length: about 9 months	Patients received diagnosis from senior consultants	Accuracy: 87.4% (C: 99.1%, $p<0.001$)<br>PPV: 74.4% (99.2%, $p<0.001$)<br>NPV: 95.0% (99.1%, $p<0.001$)	Time to diagnosis: I (2.79 min) vs. C (8.53 min), $p<0.001$	High patient satisfaction	N/A
Lindsey et al, 2018 [45]	Clinicians assisted by a DL-based model to detect	Clinicians without AI assistance	AUC: 0.967 and 0.994 on two test datasets	Sensitivity: I (91.5%) vs. C (80.8% (95% CI), $p<0.0001$	N/A	N/A

	fractures in wrist radiographs<br>Task: 300 radiographs			Specificity: I (93.9%) vs. C (87.5% (95% CI), p<0.0001<br>Misinterpretation rate: decreased by 47.0%		
Liu et al, 2020 [46]	Patients undergo colonoscopy with assistance from a CADe system<br>Length: 6 months	Patients undergo colonoscopy without AI assistance	N/A	ADR[d] and PDR[d]: increased ADR (0.39 vs. 0.24) and PDR (0.44 vs. 0.28), increased number of detected adenomas (250 vs. 144) and polyps (486 vs. 248) (p<0.001), no increase in the detection of large adenomas (ns)	N/A	N/A
Mango et al, 2020 [47]	Physicians assisted by Koios DS for Breast to make breast ultrasound lesion assessment<br>Task: 900 breast lesions	Physicians without AI assistance	Sensitivity: 0.98<br>Specificity: 0.50<br>AUC: 0.87<br>(> reader alone evaluation)	AUC: I (0.87) vs. C (0.83), p<0.0001<br>Inter-reliability (Kendall τ-b): I (0.68) vs. C (0.54), p<0.01<br>Intra-reliability improved: I (13.6%) vs. C (10.8%)	N/A	N/A
Martin et al, 2012 [48]	Patients assigned to intervention group (implementation of a complex adaptative chronic care system)<br>Length: 12 months	Patients assigned to usual care group	Sensitivity: 100%<br>PPV: 70%	N/A	ACSC[d] readmission reduced by 50%<br>Descriptive findings on care guides-supported activities and health service utilization	N/A
McCoy and Das, 2017 [49]	Implementation of InSight to predict sepsis	Pre-implementation period	N/A	N/A	Hospital mortality: decrease by 60.24% (p<0.01)<br>Hospital LOS: decrease by 9.55% (p=0.077)<br>Readmission rate: decrease by 50.14% (p<0.01)	N/A
McNamara et al, 2019 [50]	Clinicians with assistance from IBM Watson for Oncology with Cota RWE platform<br>Task: 223 patient cases	Clinicians without assistance	N/A	Decision making: no significant difference in concordance; novices were more likely to choose non-recommended options without assistance (p<0.01) and	N/A	N/A

				changed decisions in 39% cases.		
Mori et al, 2018 [51]	Real-time use of CAD during colonoscopy<br>Length: 7 months	N/A	NPV: 96.4% (best-case scenario) and 93.7% (worst-case scenario) with stained mode; 96.5% (best-case scenario) and 95.2% (worst-case scenario) with NBI	Time to diagnosis: 73 seconds for stained mode and 19 seconds for NBI mode	N/A	N/A
Nagaratnam et al, 2020 [52]	Implement e-Stroke Suite to improve mechanical thrombectomy referral pathway	N/A	N/A	N/A	One patient case illustrates how e-Stroke supports patient care and improves clinical outcomes.	N/A
Natarajan et al, 2019 [53]	Analyze the retinal images with Medios AI<br>Length: 2 months	N/A	Sensitivity: 100.0%<br>Specificity: 88.4% (higher than ophthalmologist: 85.2% sensitivity, 92.0% specificity)	N/A	N/A	N/A
Nicolae et al, 2020 [54]	Patients receive treatment planning from a ML-based prostate implant planning system<br>Length: 7 months	Conventional, manual technique	Day 30 dosimetry: ns	Planning time: I (2.38 min) vs. C (43.13 min), $p<0.05$	N/A	N/A
Park et al, 2019 [55]	Clinicians augmented with HeadXNet Model to predict intracranial aneurysms<br>Tasks: 115 examinations	Clinicians without model augmentation	N/A	Sensitivity: increased by 0.059 ($p=.01$)<br>Specificity: ns<br>Accuracy: increased by 0.038 ($p=.02$)<br>Interrater agreement (Fleiss κ) increased by 0.060 ($p=.05$).<br>Time to diagnosis: ns	N/A	N/A
Romero-Brufau et al, 2020 [56]	Implement an AI tool to improve glycemic control in patients with diabetes	N/A	N/A	Attitudes: clinical staff felt that care was better coordinated after the AI implementation ($p<0.01$).	N/A	N/A

				However, only 14% users would recommend it. The most useful aspect is team dialog prompts, and the least useful aspect was inadequate recommended interventions.		
Rostill et al, 2018 [57]	Implement Technology integrated health management (TIHM) system for dementia care<br>Length: 9 months	N/A	N/A	System evaluation: carers are willing to recommend TIHM.	Care interventions: the paper presents 3 cases in which the system led to interventions.<br>Patient evaluations: patients were willing to recommend TIHM.	N/A
Segal et al, 2014 [58]	Neurologists with assistance from SimulConsult diagnostic decision support system<br>Task: 40 patient vignettes	Neurologists without assistance	N/A	Diagnostic errors fell from 36% to 15%, and the drop was more evident among novices.<br>Diagnosis relevance increased ($p<0.0001$), but there were no significant changes in comprehensiveness.<br>Number of workup items decreased from 5.4 to 5.1 ($p=.065$).	N/A	N/A
Segal et al, 2016 [59]	Neurologists with assistance from SimulConsult diagnostic decision support system<br>Task: 8 patient vignettes	Neurologists without assistance	N/A	Diagnostic errors fell from 28% to 15% ($p<0.0001$) and the improvement was more evident among emergency medicine physicians ($p=0.013$) and novice physicians ($p=0.012$).	N/A	N/A
Segal et al, 2017 [60]	Implementation of SimulConsult diagnostic decision support system in clinical use	N/A	N/A	Perceptions: medical specialists agreed that the tool was useful and could improve workflow. However, they expressed concerns over possible legal risks.	N/A	N/A
Segal et al, 2019 [61]	Integration of MedAware (prescription error	N/A	89% alerts were accurate, 85% alerts	N/A	43% alerts changed later medical orders.	N/A

	identification and prevention systm) into clinical practice<br>Length: 1 year and 11 months		were confirmed clinically valid, 80% were considered clinically useful.			
Shimabukuro et al, 2017 [62]	Patients receive AI-generated sepsis prediction<br>Length: 3 months	Normal standard care	N/A	N/A	LOS: I (10.3 days) vs. C (13.0 days), p=0.042<br>ICU LOS: I (6.31 days) vs. C (8.4 days), p=0.03<br>In-hospital mortality: I (8.96%) vs. C (21.3%) (p=0.018)	N/A
Sim et al, 2020 [63]	Radiologists assisted by ALAND to detect malignant lung nodules on chest radiographs<br>Task: 800 radiographs	Radiologists without assistance	Sensitivity: 67.3%<br>FPPI[d]: 0.2	Sensitivity: I (70.3%) vs. C (65.1%), p<0.001<br>FPPI: I (0.18) vs. C (0.2), p<0.001<br>Decision change: 104 of 2400 radiographs were positively changed, 56 of 2400 radiographs were changed negatively.	N/A	N/A
Steiner et al, 2018 [64]	Pathologists assisted by a DL model when reviewing lymph nodes for metastatic breast cancer<br>Task: 70 digitized slides from lymph node sections	Pathologists without assistance	N/A	Sensitivity: I (91%) vs. C (83%), p=0.02<br>Average review per image: I (61s) vs. C (116s), p=0.002<br>Interpretation difficulty: pathologists perceived the image review to be easier when assistance is available (p=0.0005).	N/A	N/A
Su et al, 2020 [65]	Patients undergo colonoscopy with the assistance from an AI system<br>Length: 8 months	Patients undergo colonoscopy without the assistance from the AI system	N/A	ADR: I (0.289) vs. C (0.165), p<0.001<br>Number of adenomas per procedure: I (0.367) vs. C (0.1178), p<0.001<br>PDR: I (0.383) vs. C (0.254), p<0.001	N/A	N/A

				Number of polyps per procedure: I (0.575) vs. C (0.305), p<0.001<br>Withdrawal time: I (7.03 min) vs. C (5.68 min), p<0.001<br>Adequate bowel preparation rate: I (87.34%) vs C (80.63%), p=0.023		
Titano et al, 2018 [66]	Implement an AI system to triage cranial images<br>Task: 180 images	Standard triage workflow (human only)	N/A	Time to diagnosis: I (1.2s) vs. C (177s), p<0.0001<br>More urgent cases appeared earlier in the queue (p=0.01)	N/A	N/A
Vandenberghe et al, 2017 [67]	Use the DL-based algorithm to recognize cancer cell types and diagnose breast cancer<br>Task: 71 breast tumor resection samples	N/A	N/A	Decision concordance: 12 discordance cases were found (83% concordance rate).<br>Diagnosis modification: 8 cases were modified.	N/A	N/A
Voerman et al, 2019 [68]	Implement an antibiotic stewardship algorithm for hospitalized sepsis and lower respiratory tract infections (LRTI) patients<br>Length: two 4-year periods (2006-2009, 2010-2014)	N/A	N/A	N/A	Numbers of patients with Clostridium difficile and antibiotic resistance infections, LOS, and antibiotic use decreased (statistical significance NR).	Average total costs per patient: decreased $25,611 (49%) for sepsis and $3630 (23%) for LRTI (statistical significance NR)
Wang et al, 2019 [69]	Patients undergo colonoscopy with assistance from a polyp and adenoma detection system<br>Length: 6 months	Patients undergo colonoscopy without AI assistance	N/A	ADR: I (29.1%) vs. C (20.3%), p<0.001<br>Number of adenomas: I (0.53) vs. C (0.31), p<0.001<br>Number of diminutive adenomas: I (185) vs. C (102), p<0.001<br>Number of large adenomas: I (77) vs. C (58), p<0.001	N/A	N/A

				Number of hyperplastic polyps: I (114) vs. C (52), p<0.001		
Wang et al, 2019 [70]	Implement a system that identifies under-use of anticoagulation in 14 clinics (one clinic entered every 28 days)<br>Length: 14 months	Usual care	N/A	New anticoagulant prescriptions: I (4.1%), C (4.0%), p=0.86<br>Out of 1727 high-risk patients, 432 lacked evidence of anticoagulant prescriptions in the prior year. Pharmacists found that 17% patients (75 of 432) were potentially undertreated. The rest were excluded due to prior AF episode, documented anticoagulation refusal, and other reasons.	N/A	N/A
Wang et al, 2020 [71]	Patients undergo colonoscopy with assistance from EndoScreener<br>Length: 5 months	Patients undergo colonoscopy without EndoScreener	N/A	ADR: I (34%) vs. C (28%), p=0.030<br>PDR: I (52%) vs. C (37%), p<0.0001<br>Number of adenomas: I (1.04) vs. C (0.64), p<0.0001<br>Number of polyps: I (0.58) vs. C (0.38), p<0.0001	N/A	N/A
Wijnberge et al, 2020 [72]	Patients receive the early warning system for atrial fibrillation<br>Length: 11 months	Standard care	N/A	N/A	Median time-weighted average of hypotension: I (0.10 mm Hg) vs. C (0.44 mm Hg), p=0.001<br>Median time of hypotension per patient: I (8.0 min) vs. C (32.7 min), p<0.001<br>Treatment: ephedrine (I: 6%, C: 14%, p<0.001); vasopressors or fluids: ns<br>Time to intervention: I (53s) vs C (87s), p<0.001<br>Adverse events (I:0, C:2)	N/A

Wu et al, 2019 [73]	Implement the cataract AI referral platform for collaborative management of cataracts<br>Length: 6 months	N/A	AUC: >99%	Ophthalmologist-to-population service ratio increased by 10.2 fold compared with traditional healthcare system.	N/A	N/A
Wu et al, 2019 [74]	Patients undergo EGD with assistance of WISENSE system<br>Length: 3 months	Patients undergo EGD without assistance of WISENSE system	Accuracy: 90.40%<br>Completeness of photo-documentation: ns	Blind spot rate: I (22.46%), C (5.86%), p<0.001<br>EGD inspection time: I (5.03 min), C (4.24 min), p<0.001<br>Decreased number of ignored patients in gastric sites and in the lesser curvature of middle-upper body in forward view (p<0.001).	Adverse event: no significant adverse events.	N/A
Yoo et al, 2018 [75]	A radiologist assisted by a system for thyroid nodule diagnosis<br>Task: 50 patients undergo ultrasonography	The radiologist without system assistance	Sensitivity: 80.0%<br>Specificity: 88.1%<br>PPV: 83.3%<br>NPV: 85.5%<br>Accuracy: 84.6% (compared with radiologist alone: ns)	Compared with unassisted radiologist,<br>Sensitivity: I (92.0%), C (84.0%), p=0.037<br>Specificity: I (85.1%), C (95.5%), p=0.005<br>PPV: I (82.1%), C (93.3%), p=0.008<br>NPV: ns<br>Accuracy: ns<br>Compared with system,<br>Sensitivity: I (92.0%) vs. C (80.0%), p = 0.009<br>Specificity: ns<br>NPV: I (93.4%) vs. C (88.9%), p = 0.013<br>PPV: ns<br>Accuracy: ns	N/A	N/A

[a]N/A: not applicable

[b]I: interventional group; C: control group

[c]ns: not significant

[d]ADR: adenoma detection rate; FPPI: false-positive per image; ICU: intensive care unit; LOS: length of stay; PPV: positive predictive value; NPV: negative predictive value; PDR: polyp detection rate
